# Media Reports and Knowledge of e-Cigarette or Vaping Use-Associated Lung Injury Among Adolescents in California: Population-Based Cross-Sectional Study

**DOI:** 10.2196/69151

**Published:** 2025-07-29

**Authors:** Jijiang Wang, John Ayers, Eric Leas, Anthony Gamst, Shu-Hong Zhu

**Affiliations:** 1 Herbert Wertheim School of Public Health, University of California, San Diego, La Jolla, CA, United States; 2 Moores Cancer Center, University of California, San Diego, 9500 Gilman Drive, MC 0905, La Jolla, CA, 92093-0905, United States, 1 8583001056; 3 Qualcomm Institute, University of California, San Diego, La Jolla, CA, United States; 4 Department of Mathematics, University of California, San Diego, La Jolla, CA, United States

**Keywords:** E-cigarette or vaping use-associated lung injury (EVALI), middle and high school students, youth, risk perceptions, media, social norms, tobacco, vaping

## Abstract

**Background:**

e-Cigarette or vaping use-associated lung injury (EVALI), first reported in July 2019, caused over 2807 hospitalizations and 68 deaths by February 2020, when the outbreak subsided and the Centers for Disease Control and Prevention (CDC) stopped updating the case number. Media coverage of EVALI was extensive but not always accurate concerning the cause, which turned out to be vitamin E acetate, a compound added to certain illicit cannabis vape products. Studies have documented a significant increase in the perceived risk of vaping among the US adult population. However, research on how the EVALI outbreak influenced adolescents’ knowledge of the illness and their perception of the risk of vaping products is limited, especially those that used probability sampling of the adolescent population.

**Objective:**

This study examined knowledge of EVALI among adolescents and explored the impact of media messages on their perceptions of the condition.

**Methods:**

Archived news reports on EVALI from an online tobacco media analysis engine, Tobacco Watcher (July 2019-March 2020), and data from the California Student Tobacco Survey, a large statewide school-based survey of 8th, 10th, and 12th graders (September 2019–March 2020; N=157,499), were analyzed. Students’ awareness of EVALI and perceptions of its cause were examined in relation to their sources of information about EVALI, and their perceived risk of vaping was analyzed by their awareness of EVALI.

**Results:**

Of 19,661 news reports on Tobacco Watcher that discussed EVALI, 55.9% mentioned cannabis. Among the 157,499 middle and high school students participating in the statewide survey in California, 75% had heard about EVALI. The awareness level was similarly high for 8th, 10th, and 12th graders (75.7%, 74.6%, and 74.8%, respectively). Their primary source of knowledge about EVALI was media (63.1%), followed by parents (16.6%), teachers (8.1%), friends (7.7%), and peers (4.6%). Most students, 55%, believed nicotine was the cause of EVALI, while only 11% thought it was related to cannabis in vapes. Students who had heard about EVALI were more likely to rate vaping every day as extremely harmful than those who had not heard about it (67.8% vs 50.9%; *P*<.001).

**Conclusions:**

Most adolescents were aware of EVALI and cited media as the main source of their knowledge. The effects of extensive news coverage of EVALI have reached students as young as 8th graders. Most of those who were aware of EVALI, however, incorrectly believed that nicotine in vapes was the cause of EVALI. The actual cause—vitamin E acetate found in certain cannabis vapes—appeared to have been overlooked or not effectively communicated, especially in early media reports. Media coverage of EVALI presents a case study of the critical but complicated role of modern media in communicating health information.

## Introduction

The period from 2017 to 2019 saw a remarkable increase in vaping among American youth [1,2]. According to the National Youth Tobacco Survey, vaping prevalence among adolescents increased from 7.9% in 2017 to 20.2% in 2019 [2]. During this period, the tobacco industry dramatically increased its efforts to promote vaping [3], and public health entities countered with antivaping campaigns [4-8]. As these organized media battles were waged, an unexpected development hit the news: a vaping-related outbreak of hospitalizations and deaths. The US Centers for Disease Control and Prevention (CDC) called the new health condition e-cigarette or vaping use-associated lung injury (EVALI).

The first report of EVALI emerged in July 2019 [9]. By February 2020, when the outbreak subsided and the CDC ceased regular reporting about EVALI, 2807 hospitalizations and 68 deaths were attributed to it [10].

Vitamin E acetate, a cutting agent found in some illicit vapes containing tetrahydrocannabinol (THC, a psychoactive compound in cannabis), was eventually considered to be the cause of EVALI. However, this was not immediately clear. It was not until early September 2019 that the Food and Drug Administration (FDA) and the New York State Department of Health reported detection of high levels of vitamin E acetate in nearly all cannabis-containing vapes submitted by EVALI patients [11,12]. CDC first linked EVALI to black market THC vapes in late September [13]. CDC did not officially recognize vitamin E acetate as the primary cause of EVALI until November 2019, after confirming its presence in all tested bronchoscopy and bronchoalveolar lavage samples [14].

In the early months of the outbreak, media coverage surged. The New York Times alone published 141 EVALI-related articles in the second half of 2019 [15]. For the tobacco control field, the rising prevalence of vaping in the United States was already a controversial issue. But this level of media coverage related to vape products was unprecedented. Media reports during the period, however, often lacked precision [16,17]. Many reports at the time presented information in a way that could have led readers to associate EVALI with any vape products. Notably, the CDC’s official statements at the time used broad language when discussing potential causes of the outbreak, further blurring the distinction between illicit THC vapes and regulated nicotine products [18,19].

Multiple studies have found associations between EVALI and changes in risk perceptions and vaping behavior in the general US population [20-23], but research on its impact among adolescents remains limited. We found only one study examining EVALI-related risk perceptions among US adolescents that was based on a probability sample [24]. The prevalence of vaping among US adults dipped briefly following EVALI, then continued rising [25]. Vaping among adolescents, however, began declining after EVALI and has not returned to pre-EVALI levels [2]. Several factors may explain this trend, including school closures during the COVID-19 pandemic [26]. The impact of EVALI itself is another potential factor meriting careful examination [20,23].

The present study examined how media reports influenced adolescents’ awareness and perceptions of EVALI, which could potentially influence their vaping behavior. Media messages can influence individual knowledge and beliefs, structurally shape social norms, and eventually impact population behavior [27]. Many studies have documented the effects of pro- and antitobacco media on youth tobacco use [28-33]. However, they dealt mostly with paid advertising, whether sponsored by industry or by public health agencies [28-30,32]. Effects from earned media are harder to demonstrate, as earned health-related media coverage often follows short news cycles [34-36], making it difficult to track sustained changes in behavior, unlike paid campaigns that run over extended periods [37]. Earned media for EVALI, however, was extensive and spanned more than 7 months [38,39], so it may have had a significant impact on public perceptions of vaping [23,24,39].

A study that recruited a convenience sample of adolescents from dental clinics found that 74.2% were aware of EVALI [40]. Another study that included both adolescents and young adults in a population survey (N=3536) found that EVALI awareness was associated with a perceived risk of lung injury and the belief that vapes contain dangerous chemicals [24]. The present study investigated adolescents’ awareness of EVALI using a large representative sample (>150,000) of middle and high school students in California.

We examined awareness of EVALI among adolescents, how they first learned about the condition, their perceptions of the cause, and whether knowledge of EVALI predicted their harm perceptions of vaping. The study analyzed data from a large student survey in California conducted during and after media coverage of the outbreak. The intense media coverage of EVALI began in August 2019 and ended in January 2020 [38]. The 2019‐2020 California Student Tobacco Survey (CSTS) was fielded between September 2019 and March 20, 2020.

To verify the media cycle for EVALI coverage, we queried the database of Tobacco Watcher, a site that uses artificial intelligence to track tobacco-related media in real time [41]. Archived articles from July 2019 to March 2020 were analyzed. By examining both the student survey data and the media report data, this study aimed to address a knowledge gap on how the EVALI outbreak news reports influenced adolescents’ knowledge of EVALI and their perception of the harm of vaping.

## Methods

### Data Source

The study used data from the 2019‐2020 CSTS, a school-based survey with a large representative sample of California students in grades 8, 10, and 12 [42]. The survey used multistage sampling to select schools and then classrooms in these grades. It investigated tobacco use among adolescents through an online platform developed by the University of California, San Diego [42]. A total of 162,675 students completed the survey, which had a response rate of 68.3%. Details of the survey can be found elsewhere [42]. EVALI-related questions were added shortly after the survey cycle began, just after the peak of the outbreak. This resulted in 2353 students (1.4% of the total sample) not being asked the EVALI questions and being excluded from the analysis. Another 2823 students did not answer the EVALI questions, leaving an effective sample size of 157,499 participants.

Media data were obtained from Tobacco Watcher, a global media monitoring platform that automatically identifies and categorizes tobacco-related news reports from over 595,000 sources [41]. The system uses an extensive list of more than 1000 tobacco-related keywords in 21 languages (English, Arabic, Bengali, Chinese, French, German, Hindi, Indonesian, Italian, Japanese, Korean, Polish, Portuguese, Russian, Spanish, Tagalog, Tamil, Thai, Turkish, Ukrainian, and Urdu) to identify relevant news articles. This list includes general terms (eg, “tobacco,” “smoking,” and “nicotine”), regulatory and policy-related phrases (eg, “tobacco tax” and “flavor ban”), specific product categories (eg, “e-cigarette,” “hookah,” and “snus”), and brand names (eg, “Marlboro” and “JUUL”). First, the database of news sources is searched using these keywords, and an article is considered for inclusion in Tobacco Watcher if it contains at least one keyword. About 50,000 articles per day are captured during this phase. Second, articles are filtered for tobacco relevance using a binary classifier that determines whether they are tobacco-related or not. The platform uses supervised machine learning, specifically a fine-tuned Robustly Optimized Bidirectional Encoder Representations from Transformers Approach (RoBERTa) transformer model, to classify news articles. The model was trained on a corpus of labeled articles annotated by human reviewers. It analyzes both lexical content and semantic context, allowing it to detect nuanced references to tobacco-related topics even when keyword matches are ambiguous or absent. About 600 articles per day are labeled as tobacco-related and included on the Tobacco Watcher website. All tobacco-relevant articles were downloaded and archived and are available to the Tobacco Watcher analysis engine for analysis.

### Measures

#### Awareness and Knowledge of EVALI

The survey asked students, “Have you heard about people getting sick or even dying from using vapes?” Those who answered “yes” were asked, “Where did you FIRST hear about it?” with options including “parents,” “teachers,” “friends,” “peers,” and “media.” They were also asked, “What do you think is in the vapes that is the MOST LIKELY cause of illness or death?” Options included “nicotine,” “marijuana,” “flavoring,” “other,” and “I don’t know.” (Can be found in Multimedia Appendix 1 for the survey questions). Students who selected “other” were asked to enter the perceived cause in an open-text field, which was later coded independently by 2 staff researchers in Zhu’s lab. The purpose of this coding process was to identify terms that could be considered the cause of the illness, including vitamin E acetate. These responses were grouped into one category in the analysis and were labeled as “Other chemicals” in the tables presented in the results section. The agreement between the 2 coders was 99.8% for these terms which were eventually grouped into the “Other chemicals” category. The minor disagreement between the coders was resolved by Dr Zhu.

#### Vaping Status

In separate questions, the survey asked students about their nicotine and marijuana vape use in the last 30 days. Based on their answers, students were categorized as vaping both marijuana and nicotine, vaping marijuana only, vaping nicotine only, or vaping neither marijuana nor nicotine.

#### Harm Perception

The survey also asked, “How harmful do you think these products are if a person uses them EVERY DAY?” and “How harmful do you think these products are if a person uses them SOME DAYS?” A 5-point Likert scale from “not at all harmful” to “extremely harmful” was used. For analysis, responses were grouped into 2 categories, “extremely harmful” and “not extremely harmful,” for the 2 questions, respectively.

#### Demographics and Other Variables

Other variables included grade (8, 10, and 12), gender (male, female, other, declined to answer), and race/ethnicity. Self-reported race/ethnicity was categorized as non-Hispanic White, Hispanic, non-Hispanic African American or Black, non-Hispanic Asian, non-Hispanic American Indian/Alaska Native, non-Hispanic Native Hawaiian or other Pacific Islander, non-Hispanic other, and non-Hispanic multiracial, following the methodology used by the National Youth Tobacco Survey [43]. Parental education was assessed by asking whether either parent had a college degree.

### Data Analysis

#### Media Report Data Analysis

We searched the Tobacco Watcher archive (including>1,000,000 articles) using its “analyses” function for articles containing “vaping” and “outbreak” or “illness” to identify EVALI news reports. We further identified the subset of reports mentioning “marijuana,” “cannabis,” or “THC,” indicating reports potentially linking the outbreak to cannabis products. We queried the system from July 25, 2019, when the first EVALI report was archived, to March 20, 2020, the end of the 2019‐2020 CSTS. The study reports any week when more than 100 EVALI news reports were published.

#### Survey Data Analysis

Descriptive percentages and 95% CIs were calculated for prevalence estimates of EVALI awareness, source of EVALI information, perceived cause of EVALI, and perceived harm of vaping [44]. Between-group differences were examined with the Wald chi-square test. Sample weights were used to account for the complex sampling design, nonresponse adjustment, and student enrollment. The Finite Population Correction Factor was applied for narrower CI estimation when more than 5% of the target population in each region was surveyed. Complex survey procedures in SAS 9.4 (SAS Institute) were used for the analysis, with consideration of cluster effects within sampling strata and participating schools.

This study was a secondary analysis of existing data from an anonymous survey and online news articles and was covered by the University of California, San Diego, Human Research Protection Program (#170787).

### Ethical Considerations

Parental or guardian consent—either passive or active—was obtained in accordance with the specific requirements of each participating school. Student data were collected anonymously. Upon survey completion, each school received a US $500 gift card; student participants did not receive individual compensation. This study was a secondary analysis of existing data sets of anonymous surveys and was covered by the University of California, San Diego, Human Research Protection Program (#170787).

## Results

**Figure 1.**
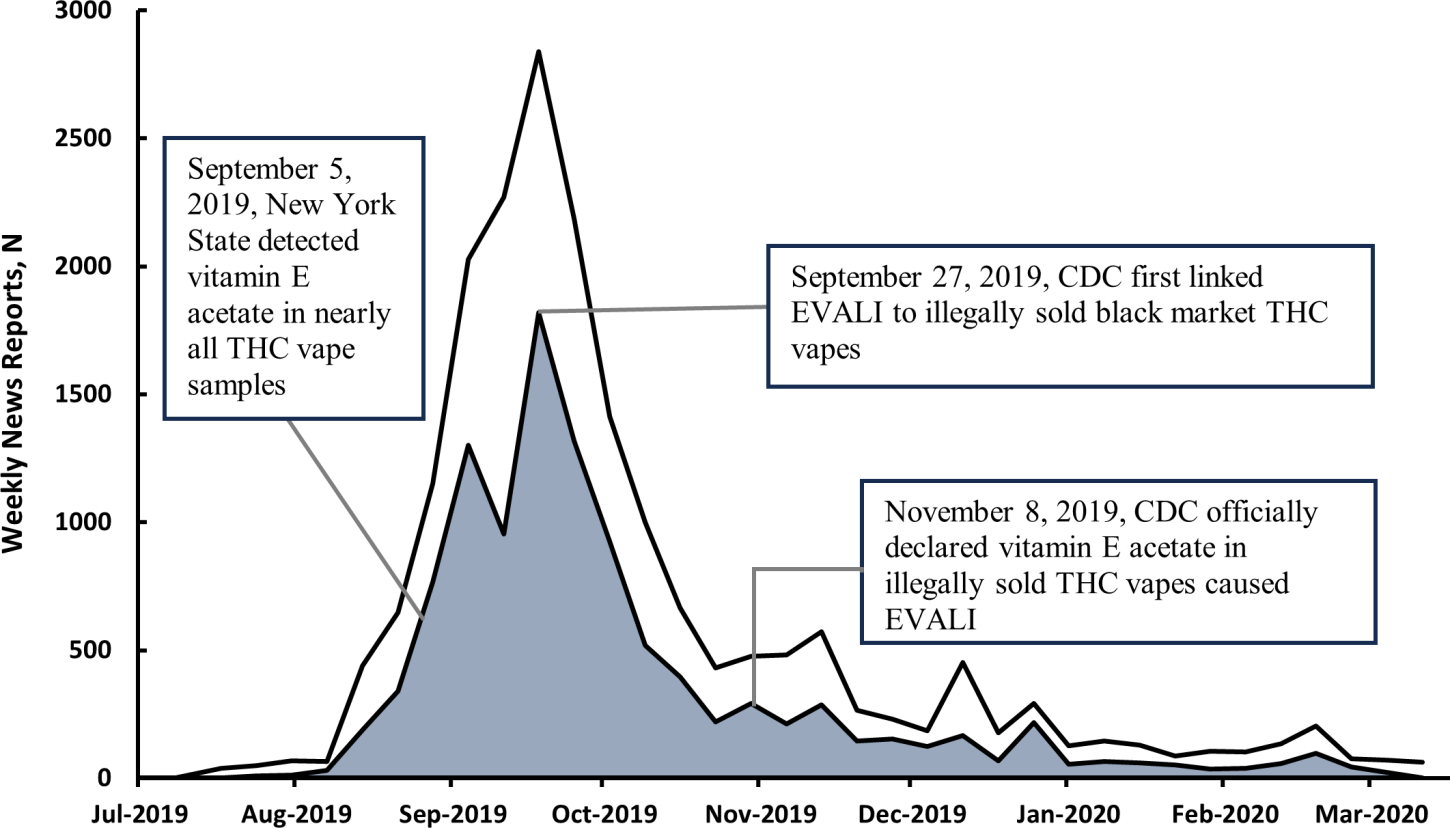
Weekly time series in news reports on the EVALI outbreak. This time series represents the number of news reports that potentially mentioned the EVALI outbreak (ie, articles relevant to tobacco from TobaccoWatcher.org that included the keywords “vaping” and “outbreak” or “illness.”) The shaded region represents the subset of articles mentioning “marijuana,” “cannabis,” or “THC.” All data are presented in weekly bins (eg, June 1‐7). CDC: Centers for Disease Control and Prevention; EVALI: e-cigarette or vaping use-associated lung injury; THC: tetrahydrocannabinol.

Figure 1 presents the number of EVALI-related news reports from Tobacco Watcher. From July 25, 2019, to March 20, 2020, 19,661 such reports discussed EVALI. The outbreak first garnered over 100 reports (specifically, 439) the week of August 18, 2019 and increased to a peak of 2840 the week of September 22, 2019. Thereafter, the weekly numbers declined but did not return below 100 until the week of January 26, 2020.

A total of 2 dates in Figure 1 deserve special mention, September 27, 2019, when the CDC first linked cannabis vapes to EVALI in its press releases, and November 8, 2019, when the CDC declared vitamin E acetate the cause of EVALI [13,14]. Figure 1 shows media coverage peaking in late September 2019 and then gradually declining. Of all the media reports in Figure 1, 44.8% appeared by September 27, 2019, and 79.2% by November 8, 2019.

The shaded area in Figure 1 represents EVALI reports that mentioned marijuana/cannabis. Among all EVALI reports, 55.9% mentioned this substance. There were 2 spikes in the trend, in the week after September 5, 2019 (Thursday), when the New York State Department of Health and the FDA announced that vitamin E acetate was the likely cause of EVALI, and on September 27, when the CDC first linked illicit THC vapes to EVALI.

Table 1 presents the percentage of students who had heard of EVALI. Awareness was high overall, at 75%, and among students in different grades. Students in grade 8 were as aware of EVALI as those in grade 12 (75.7% and 74.8%, respectively). Awareness was high across other demographic dimensions. There was some variation across gender, ethnicity, and parental education, but most students in each subgroup had heard about EVALI.

**Table 1.** e-Cigarette or vaping use-associated lung injury awareness among middle and high school students in California, 2019‐2020.

Demographics	Overall,(N=157,499), % (95% CI)	Grade 8, (N=11,771), % (95% CI)	Grade 10, (N=78,830), % (95% CI)	Grade 12, (N=66,898), % (95% CI)
Overall	75.0 (73.8-76.3)	75.7 (72.6-78.8)	74.6 (73.5-75.7)	74.8 (73.6-76.0)
Gender				
Male	73.6 (72.2-75.0)	74.5 (71.1-77.9)	72.9 (71.7-74.1)	73.4 (72.1-74.7)
Female	78.3 (77.0-79.5)	78.1 (75.1-81.1)	78.4 (77.3-79.5)	78.3 (77.0-79.6)
Other	60.9 (57.9-64.0)	66.7 (59.0-74.4)	58.2 (55.0-61.3)	57.4 (54.1-60.7)
Declined to state	57.8 (54.6-60.9)	67.7 (60.0-75.4)	54.6 (51.3-57.9)	49.3 (46.3–52.4)
Race/ethnicity				
Non-Hispanic White	86.0 (84.9-87.1)	84.9 (82.3-87.6)	86.1 (84.9-87.2)	87.2 (86.1-88.2)
Non-Hispanic Black	65.1 (62.3-67.9)	65.0 (58.4-71.7)	63.4 (60.4-66.5)	66.9 (63.5-70.3)
Hispanic	71.9 (70.7-73.0)	73.1 (70.1-76.1)	71.5 (70.5-72.4)	71.0 (69.8-72.3)
Non-Hispanic Asian	76.8 (75.3-78.2)	78.6 (75.1-82.0)	76.7 (75.0-78.3)	75.5 (73.8-77.2)
Non-Hispanic AI/AN^a^	57.6 (49.6-65.6)	53.3 (34.9-71.8)	58.0 (51.1-64.9)	64.8 (57.2-72.4)
Non-Hispanic NHOPI^b^	62.1 (56.6-67.7)	70.7 (55.9-85.5)	58.4 (52.4-64.4)	58.2 (53.0-63.3)
Non-Hispanic other	65.1 (61.7-68.4)	69.8 (63.3-76.3)	62.8 (59.1-66.4)	58.3 (54.4-62.3)
Non-Hispanic multiracial	78.2 (75.8-80.6)	76.0 (70.8-81.3)	79.5 (78.0–81.1)	80.4 (78.8-82.0)
Parental education				
College degree	82.1 (81.0-83.3)	81.8 (79.1-84.6)	82.0 (81.0-83.1)	82.6 (81.5-83.7)
Some college or lower	71.7 (70.5-72.8)	72.6 (69.0-76.3)	71.3 (70.3-72.2)	71.4 (70.3-72.6)
Unknown	64.8 (63.0-66.7)	68.3 (64.7-71.9)	63.1 (61.6-64.6)	57.4 (55.3-59.5)

a AI/AN: American Indian/Alaska Native.

b NHOPI: Native Hawaiian or other Pacific Islander.

Table 2 shows how students reported learning of EVALI. The overwhelming majority, 63.1%, first heard about EVALI from a media source. Parents were the second-most likely source (16.6%), followed by teachers (8.1%), friends (7.7%), and peers (4.6%).

**Table 2.** Initial source of e-cigarette or vaping use-associated lung injury information among middle and high school students in California, 2019‐2020.

Source of information: learned EVALI^a^ from:	Overall (N=157,499), % (95% CI)	Grade 8 (N=11,771), % (95% CI)	Grade 10 (N=78,830), % (95% CI)	Grade 12 (N=66,898), % (95% CI)
Media	63.1 (62.2-64.0)	55.9 (53.7-58.1)	62.6 (61.9-63.3)	71.3 (70.6-72.0)
Parents	16.6 (15.8-17.4)	24.6 (22.8-26.4)	15.6 (15.2-16.0)	9.1 (8.7-9.4)
Teachers	8.1 (7.4-8.7)	8.6 (6.9-10.3)	8.9 (8.3-9.4)	6.7 (6.1-7.2)
Friends	7.7 (7.2-8.1)	6.7 (5.6-7.8)	8.0 (7.7-8.3)	8.3 (7.9-8.7)
Peers	4.6 (4.4-4.8)	4.2 (3.6-4.8)	5.0 (4.8-5.2)	4.6 (4.4-4.9)

a EVALI: e-cigarette or vaping use-associated lung injury.

The likelihood that students had first heard about EVALI from a media source increased with age. But even in grade 8, most heard about EVALI from the media (55.9%). The likelihood of hearing about EVALI from parents was higher for younger students than for older students. In grade 8, 24.6% learned of EVALI from their parents, compared to 9.1% in grade 12 (*P*<.001).

Table 3 shows what students believed caused EVALI. Overall, most who had heard about the condition believed nicotine was the cause (55.0%). More than 1 in 5 (22.1%) said they did not know. Marijuana was chosen by 11.1%, followed by other chemicals (4.7%). Similar percentages thought flavorings (3.5%) or other things (3.6%) were the cause.

**Table 3.** Perceived cause of e-cigarette or vaping use-associated lung injury among middle and high school students in California, 2019‐2020.

Variables	N	Perceived cause of EVALI^a^					
		Nicotine, % (95% CI)	Marijuana, % (95% CI)	Other chemicals, % (95% CI)	Flavoring, % (95% CI)	Other, % (95% CI)	Don’t know, % (95% CI)
Overall	119,415	55.0 (54.1-55.9)	11.1 (10.7-11.6)	4.7 (4.4-5.1)	3.5 (3.3-3.7)	3.6 (3.4-3.8)	22.1 (21.5-22.7)
Learned EVALI from							
Media	78,100	55.6 (54.5-56.6)	9.7 (9.3-10.2)	5.6 (5.2-6.0)	3.4 (3.2-3.6)	3.9 (3.7-4.1)	21.8 (21.0-22.5)
Parents	15,881	55.4 (53.8-57.0)	13.9 (12.6-15.1)	2.7 (2.3-3.1)	2.7 (2.3-3.0)	2.5 (2.1-2.9)	22.9 (21.8-23.9)
Teachers	10,067	53.0 (50.8-55.1)	14.1 (13.0-15.1)	3.0 (2.6-3.5)	3.8 (3.2-4.5)	3.0 (2.2-3.8)	23.1 (21.5-24.7)
Friends	9,512	54.1 (52.3-55.9)	13.1 (11.9-14.2)	3.8 (3.2-4.3)	5.2 (4.5-6.0)	3.8 (3.2-4.4)	20.0 (18.6-21.5)
Peers	5,752	50.1 (47.3-52.9)	12.2 (10.4-14.0)	4.8 (3.9-5.7)	4.3 (3.4-5.2)	3.9 (3.0-4.8)	24.7 (22.8-26.6)
Vaping status							
Exclusive nicotine only	3,170	42.2 (39.4-45.1)	17.2 (15.2-19.2)	9.0 (7.5-10.4)	6.5 (5.0-7.9)	5.2 (3.7-6.7)	19.9 (17.6-22.3)
Exclusive marijuana	3,693	52.3 (49.1-55.4)	7.3 (5.9-8.8)	10.7 (9.2-12.2)	5.6 (4.3-6.9)	5.6 (4.8-6.5)	18.5 (16.1-20.8)
Both	5,872	43.8 (41.8-45.9)	13.0 (11.6-14.4)	10.5 (9.3-11.8)	7.9 (6.8-9.1)	7.6 (6.2-8.9)	17.1 (15.7-18.5)
Neither	106,670	55.8 (54.9-56.7)	11.0 (10.5-11.5)	4.2 (3.9-4.5)	3.2 (3.0-3.4)	3.3 (3.1-3.5)	22.4 (21.8-23.1)

a EVALI: e-cigarette or vaping use-associated lung injury.

Beliefs about the cause of EVALI did not differ much by the source from which students first learned about the condition. Over half believed nicotine was the cause, regardless of where they first learned of EVALI. There were minor variations. If students first learned of EVALI from peers, they were less likely to believe nicotine was the cause. But even in this group, nicotine was believed to be the cause by 50.1%.

Interestingly, if students first learned of EVALI from the media, they were less likely to believe marijuana was the cause of it. Among those who first learned of EVALI from the media, 9.7% thought marijuana was the cause, significantly lower than those who heard about it from other sources (12.2%‐14.1%). On the other hand, students who learned of EVALI from the media were more likely to choose “other chemicals” as the cause of EVALI (5.6%) than those who heard about it from other sources (2.7%‐4.8%). Thus, if the proportion of students believing marijuana was the cause was combined with the proportion believing certain “chemicals” were the cause, the differences between sources disappear. The sums of these 2 proportions were 15.3%, 16.6%, 17.1%, 16.9%, and 17.0% for media, parents, teachers, friends, and peers, respectively.

Table 3 also shows that perceptions varied by vaping status. Those who vaped nicotine, whether nicotine alone or nicotine and marijuana, were less likely to believe nicotine caused EVALI (42.2% and 43.8%, respectively) than either those who vaped marijuana only or those who did not use any vaping products (52.3% and 55.8%, respectively). The former 2 groups were more likely to consider marijuana the cause of EVALI than the latter 2 groups (17.2% and 13.0%, respectively, vs 7.3% and 11.0%, respectively). The rank order, however, was similar across vaping status. All subgroups, regardless of vaping status, were more likely to indicate nicotine as the cause, with “I don’t know” as the next most frequent option.

Table 4 presents students’ awareness and knowledge of EVALI based on when they took the survey. As shown in Figure 1, most media coverage was before November 2019. We divided respondents into 3 periods based on when they took the survey. The first period was before when the CDC declared vitamin E acetate as the cause of EVALI (November 8, 2019). The other 2 periods divided the remainder of the study period into roughly equal halves. Awareness of EVALI remained high across all 3 periods, with a slight decrease in Period 3 (72.9%) compared to Period 2 (77.3%) and Period 1 (78.8%).

**Table 4.** Perceived cause of e-cigarette or vaping use-associated lung injury among middle and high school students in California by period of survey, 2019‐2020.

Variables	Period 1	Period 2	Period 3
	September 30, 2019, to November 7, 2019 (N=15,753), % (95% CI)	November 8, 2019, to January 13, 2020 (N=49,610), % (95% CI)	January 14, 2020, to March 20, 2020 (N=92,136), % (95% CI)
Heard of EVALI^a^	78.8 (74.3-83.2)	77.3 (75.1-79.5)	72.9 (71.4-74.4)
Perceived cause of EVALI^b^			
Nicotine	54.4 (51.0-57.8)	54.5 (52.8-56.2)	55.4 (54.4-56.4)
Marijuana	10.9 (9.0-12.8)	11.3 (10.7-11.9)	11.1 (10.4-11.7)
Other Chemicals	5.0 (3.9-6.1)	4.6 (3.8-5.3)	4.8 (4.2-5.3)
Flavoring	3.8 (3.0-4.5)	3.7 (3.3-4.1)	3.3 (3.1-3.6)
Other	3.8 (3.1-4.5)	3.7 (3.3-4.0)	3.5 (3.3-3.7)
Do not know	22.1 (19.9-24.4)	22.3 (21.2-23.3)	21.9 (21.2-22.7)

a EVALI: e-cigarette or vaping use-associated lung injury. Period 1: n=15,753; period 2: n=49,610; and period 3: n=92,136.

b Period 1: n=12,430; period 2: n=38,668; and period 3: n=68,317.

Table 4 also shows that there was little difference in students’ perceived cause of EVALI across the 3 periods. Students surveyed in the first period had similar responses regarding the cause of EVALI as those surveyed later when the media cycle for EVALI had mostly ended. Most in the later periods still attributed EVALI to nicotine.

**Figure 2.**
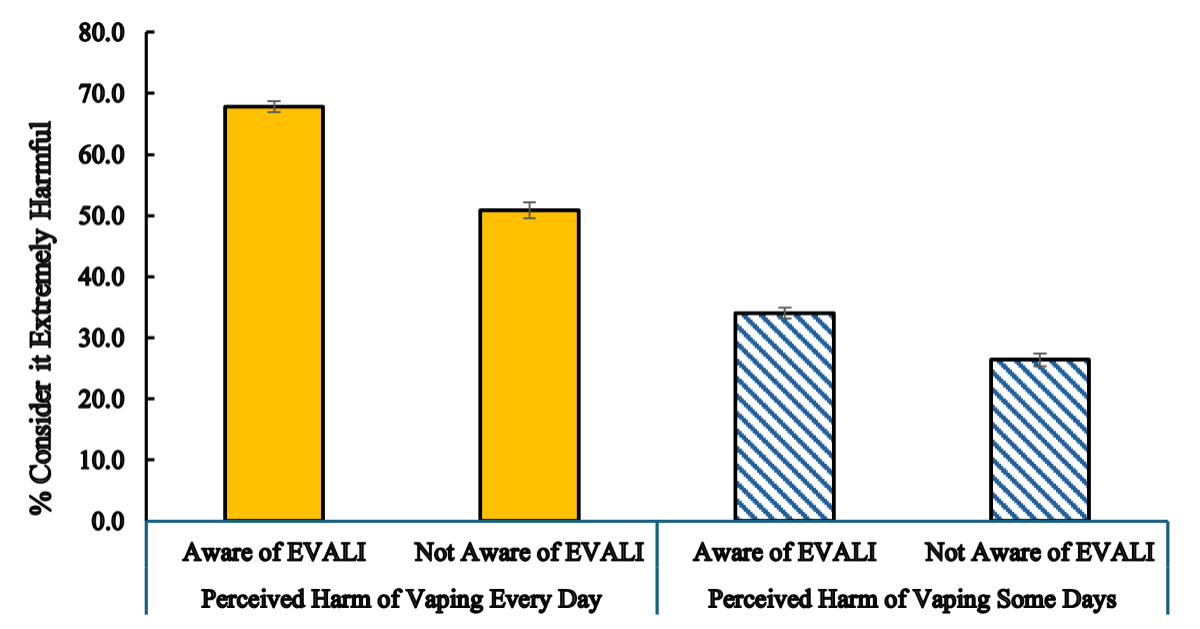
Perceived harm of vaping every day and some days among middle and high school students in California, 2019‐2020. EVALI: e-cigarette or vaping use-associated lung injury.

Figure 2 shows students’ perceived harm of vaping by awareness of EVALI. The 2 bars at the left show that those who had heard about EVALI were more likely to rate vaping every day as extremely harmful than those who had not heard about EVALI (67.8% vs 50.9%; *P*<.001). Their perception of the harm of vaping some days follows the same pattern: those who had heard about EVALI were more likely to rate vaping some days extremely harmful than those who had not (34.0% vs 26.4%; *P*<.001).

## Discussion

### Principal Findings

This study, with a large representative sample of adolescents, found that youth awareness of EVALI in California during and soon after the outbreak was high, at 75%. The media was the primary source of this awareness, including among younger students. Most respondents believed nicotine was the cause of EVALI injuries and deaths. Those who had heard about EVALI were more likely to think vaping was extremely harmful than those who had not. These results suggest that knowledge of EVALI likely had a substantial impact on adolescents’ perceptions of vaping products.

Nearly two-thirds of participants said they first learned about EVALI from the media. This confirms the results of an earlier study with adolescents and young adults [24]. There are likely at least 2 reasons for the high level of attribution to the media. First, media reports for EVALI were voluminous (eg, 141 articles in the New York Times) and the coverage lasted for an extended time. While the media cycle for EVALI has not been formally defined, this study found that from mid-August 2019 to the end of January 2020, there were at least 100 new articles per week about EVALI online, or nearly 20,000 articles total. Media coverage of a single health topic over such an extended period is rare [34,36]. Second, news about EVALI likely spread quickly from traditional media channels to social media platforms. Many studies have reported the amplification of news reports from traditional media channels to social media [38,45,46]. It helps explain why students identified media as their first source of information for EVALI.

Adolescents in this study may have had both direct and indirect exposure to news reports on EVALI. The Social Diffusion Model posits that media activities influence populations through direct exposure, institutional diffusion, and interpersonal communication [47]. As an example of institutional diffusion, soon after news of EVALI broke, the governor of California allocated additional money for paid media to combat vaping among adolescents [48]. Regarding interpersonal communication, many students heard about EVALI from interpersonal discussions with parents, teachers, friends, and peers (Table 2). Moreover, beliefs about the cause of EVALI were distributed similarly among students who learned of EVALI from the media and those who learned of it from interpersonal sources, suggesting substantial overlap in messaging (Table 3).

The study found that 55% of students chose nicotine as the cause of EVALI when presented with multiple options. Only 11% chose cannabis, which would be more directly related to the real cause of EVALI. This finding merits comments. First, nicotine was not the cause of EVALI. Second, this incorrect attribution was likely caused by how the media reported the outbreak which generally linked EVALI to e-cigarettes, a term that is mostly used for nicotine vapes [49]. Third, the attribution, whether correct or not, may have contributed to the decline in nicotine vaping among youth post EVALI [2].

The CDC declared the true cause of EVALI was vitamin E acetate, a substance added to some cannabis vapes sold illegally in certain markets. However, the declaration came in November 2019. The press releases by the CDC in early September avoided making a clear identification of the cause [50,51], even though evidence linking EVALI to cannabis vapes in August 2019 and the detection of vitamin E acetate in THC vape products used by EVALI patients in early September 2019 [12,52]. In the early phase of the outbreak, the CDC generally recommended avoiding the use of all vape products, while mentioning that cannabis products are found in the mix of EVALI clinical reports [50,51]. CDC first directly linked cannabis vapes to EVALI on September 27 and did not officially declare vitamin E acetate as the cause until November 8, 2019 [13,14]. As shown in Figure 1, about 45% of media reports about EVALI on the internet appeared before September 27, and almost 80% before November 8.

It appears that the message from the early media reports on EVALI set the tone for almost the entire period of the outbreak [39]. For example, even though a portion of reports did mention marijuana/cannabis, the proportion did not change much following the CDC’s later releases containing a discussion of the true cause of EVALI. News outlets could have paid greater attention to the announcement on September 5, 2019, by the New York State Department of Health identifying vitamin E acetate as the likely cause of EVALI [12]. By relying too heavily on early CDC press releases, media outlets may have led people to believe that the use of any vaping products could lead to EVALI [15,39]. The ambiguous name of EVALI, which includes the phrase “e-cigarette and vape use,” implies that [53]. The fact that more than 40% of nearly 20,000 reports on EVALI, including those appearing after September, did not mention marijuana/cannabis suggests that the narrative about EVALI was largely set by early September (Figure 1). Table 4 shows that the public attention to EVALI faded over time, but there was little change in students’ attribution of the cause of EVALI after November 2019, when the CDC officially declared vitamin E acetate as the cause of EVALI.

Because most survey respondents reported media as their initial source of information about EVALI, how the media reported on it likely helped lead to a misperception among youth that nicotine was the cause of the outbreak [22,53,54]. Only the direct experience of using nicotine vapes seemed to decrease this misattribution. As shown in Table 3, current users of nicotine-containing vapes were less likely to attribute EVALI to nicotine. But even in this group, nicotine was considered a more likely cause than cannabis or other chemicals.

It is worth mentioning that a large proportion of adults in a population-based study also attributed EVALI to nicotine, even a year after the outbreak [22]. Unlike adolescents in this study, however, current vapers in the adult study were more likely to attribute EVALI to cannabis than to nicotine, while non-vapers were more likely to attribute it to nicotine than to cannabis.

Adolescents’ knowledge of EVALI predicts their perceptions of the risk of vaping. Those who had heard about the condition were more likely to consider vaping extremely harmful than those who had not. This finding helps explain the results of other studies showing that EVALI led to an overall increase in the perceived risk from vaping in the United States [1,23,24]. The change in perception may further impact intention and behavior; adolescents who have not used vapes may be less likely to use them in the future, and those who currently use them may be more likely to stop if their perception of harm increases [24]. Both intentions to use and quitting behaviors may impact future vaping prevalence. Vaping prevalence among US adolescents has indeed declined since EVALI [2]. There may be multiple reasons for this, including school closures due to COVID-19, which occurred soon after EVALI. The outbreak of EVALI, however, became an inflection point for adolescent vaping behavior in the United States. The prevalence of vaping among US adolescents dropped dramatically from 20.2% in 2019 to 13.3% in 2020, and further to 5.9% in 2024, a trend not observed among their European counterparts, where few EVALI cases were reported [55-57]. In the United Kingdom, for example, adolescent vaping rates remained relatively stable before and after the EVALI outbreak (4.4% in 2019 vs 4.1% in 2020), then increased to 7.2% in 2024. By examining the connection between the extensive media coverage of EVALI in the United States and adolescents’ knowledge and perceptions related to it, this study helps illuminate the change in vaping prevalence among US adolescents.

One might be tempted to argue that the impact of media reports on adolescent vaping was good for public health even if messaging about the cause was often inaccurate. However, if the messaging is considered misleading, it can erode trust in public health authorities [53]. Moreover, misinformation related to the cause of EVALI could have negative effects that may not immediately be obvious. For example, it is unclear how much the misattribution of the cause of EVALI increased the probability of adolescents using cannabis vapes instead of nicotine vapes, especially given that they already considered cannabis less risky than nicotine [58].

### Limitations

This study has limitations. First, Tobacco Watcher only tracked the use of terms in media reports. It did not provide a detailed analysis of the contents. However, a simple count of how often marijuana/cannabis was mentioned in the reports is consistent with the findings of other studies that examined the content in greater detail [39]. Second, the survey did not provide an option for vitamin E acetate as a potential cause of EVALI. The survey questions were developed before vitamin E acetate was identified by the CDC as a possible cause of the illness. Third, half of the survey was fielded during the EVALI outbreak (and about 1.4% of students were excluded from the analysis because they were surveyed before the EVALI questions were added to the survey). Students’ media exposure, awareness of EVALI, and beliefs about the cause may have changed over time. Even though the analysis did not find a significant change in their attribution during the survey period, suggesting the dominant role of early reports on EVALI in shaping their views, it is not clear how long the specific knowledge of EVALI would last. Fourth, the study was conducted in California, which mounted an additional campaign against vaping on top of campaigns organized by federal agencies and earned media reports [8]. This may have elevated adolescents’ perception of the risk of vaping. Fifth, the main measures of this study, awareness of EVALI, risk perception of vaping, and vaping behavior, are based on adolescents’ self-reports, which have their limitations. Finally, the term “media” is used broadly in the study, encompassing both traditional media sources and social media platforms. This broad categorization limits the ability to distinguish the specific influence of different types of media on participants’ knowledge and perceptions.

### Conclusions

In summary, the study highlights the critical role of earned media reports in disseminating information about the EVALI outbreak. While other communication strategies can raise awareness of the risk of using addictive products, earned media in this case played a leading role in raising EVALI awareness among youth and likely shaped their perceptions of it, which could influence their future vaping behaviors. The inaccuracy of a substantial proportion of media reports remains an important case study. The potential for health communication through earned media to influence adolescent behavior in this era of diverse media channels deserves more investigation.

## Supplementary material

10.2196/69151 Multimedia Appendix 1 Selected questions from the California Student Tobacco Survey 2019-2020 Survey.

## Abbreviations

CDC: Centers for Disease Control and Prevention
CSTS: California Student Tobacco Survey
EVALI: e-cigarette or vaping use-associated lung injury
FDA: Food and Drug Administration
RoBERTa: Robustly Optimized Bidirectional Encoder Representations from Transformers Approach
THC: tetrahydrocannabinol
