# Supplementary material for: Media Reports and Knowledge of e-Cigarette or Vaping Use-Associated Lung Injury Among Adolescents in California: Population-Based Cross-Sectional Study
Source: J Med Internet Res. 2025 Jul 29;27:e69151. doi: 10.2196/69151 (PMC12306916; doi:10.2196/69151)
Supplement: Multimedia Appendix 1 [file jmir-v27-e69151-s001.docx]

**Appendix 1.** Selected questions in the CSTS 2019-2020 survey that were used in the analysis for this paper

**[Vape use questions]**

15.

A. Have you EVER vaped?

A. Yes

B. No

B. Have you vaped in the LAST 30 DAYS?

A. Yes

B. No

16. You said you vaped. Which of the following have you vaped in the LAST 30 DAYS?

A. Nicotine (with or without flavor)

A. Yes

B. No

B. Marijuana (wax, oil, THC, or CBD)

A. Yes

B. No

C. Just flavoring (without nicotine, marijuana, or another drug)

A. Yes

B. No

**[EVALI questions]**

DC2. Have you heard about people getting sick or even dying from using vapes?

a) Yes

b) No

DC3. Where did you FIRST hear about it?

a) Parents

b) Teachers

c) Friends

d) Peers

e) Directly from news media (e.g., TV, radio, internet)

DC4. What do you think is in the vapes that is the MOST LIKELY cause of illness or death? (Select ONLY one)

a) Nicotine

b) Marijuana (wax, oil, THC, or CBD)

c) Flavoring

d) Other [open text] _____

e) I don’t know

**[Harm perception questions]**

100. How harmful do you think these products are if a person uses them EVERY DAY?

b) Use vapes with nicotine

A. 1 Not at all harmful

B. 2

C. 3

D. 4

E. 5 Extremely harmful

101. How harmful do you think these products are if a person uses them SOME DAYS, but not every day?

b) Use vapes with nicotine

A. 1 Not at all harmful

B. 2

C. 3

D. 4

E. 5 Extremely harmful
